# Concomitant Atrial Fibrillation Ablation and Left Atrial Appendage Occlusion: Is One-Stop the Right Stop?

**DOI:** 10.1016/j.jacadv.2026.103166

**Published:** 2026-08-19

**Authors:** Paschalis Karakasis

**Affiliations:** Second Department of Cardiology, Aristotle University of Thessaloniki, General Hospital Hippokration, Greece

Sawalha et al^1^ should be congratulated for providing a large, contemporary evaluation of concomitant atrial fibrillation (AF) ablation and left atrial appendage occlusion (LAAO). In 1,899 propensity-matched pairs, the authors observed similar coded rates of mortality, stroke, pericardial complications, device-related thrombus, and peridevice leak through 1 year. These data are reassuring and complement randomized evidence supporting the feasibility of a “one-stop” strategy.^2^ Nevertheless, several considerations temper the inference that concomitant treatment is as safe and effective as isolated LAAO.

First, the absence of statistical significance does not establish equivalence. No noninferiority margin or power calculation was specified, and the CIs remain compatible with clinically important harm: the upper bounds permit a 2.47-fold increase in 7-day stroke, a 2.57-fold increase in 90-day device-related thrombus, and a 2.32-fold increase in 90-day mortality. The most defensible interpretation is therefore that no excess-risk signal was detected, rather than that equivalent safety was demonstrated. The Central Illustration^1^ extends the conclusion further by describing the combined strategy as equally effective, although arrhythmia recurrence, AF burden, bleeding, quality of life, and repeat ablation were not evaluated.

Second, LAAO alone is not the clinically decisive comparator for patients who already have indications for both procedures. This comparison estimates the incremental risk of adding ablation among selected LAAO recipients, but it does not determine whether concomitant treatment is preferable to a staged strategy. OPTION showed favorable outcomes with LAAO after ablation and reassuring findings within both concomitant and sequential strata; however, procedural timing itself was not randomized.^2^ A dedicated concomitant-vs-staged randomized comparison remains necessary.

Selection is also difficult to neutralize fully. In the contemporary NCDR (National Cardiovascular Data Registry) LAAO Registry, only 1.9% of 96,968 patients underwent concomitant ablation; these patients were younger, had lower CHA_2_DS_2_-VASc scores, and less prior bleeding or fall risk.^3^ Propensity matching cannot capture all such channeling, including frailty, AF phenotype, operator expertise, and institutional workflow.

Third, the unavailable procedural variables are likely effect modifiers rather than merely descriptive omissions. COMBINATION (Strategy optimization for combined procedure of left atrial appendage closure and catheter ablation in patients with atrial fibrillation) demonstrated superior event-free survival with an occlusion-first rather than ablation-first sequence, emphasizing that same-day procedures are not homogeneous.^4^ Device type, ablation energy, imaging modality, and postprocedural antithrombotic therapy may also materially influence leak, thrombus, and bleeding. Contemporary evidence suggests meaningful outcome differences across post-LAAO antithrombotic regimens.^5^ Moreover, device leak and thrombus are surveillance-dependent endpoints; without protocolized transesophageal echocardiography or computed tomography, low diagnosis-code rates may reflect underdetection rather than true absence.

Thus, this study by Sawalha et al^1^ importantly supports real-world feasibility and provides no evidence of a major early safety penalty. Its findings should encourage prospective evaluation, but not yet establish procedural equivalence. Future studies should compare concomitant with staged care, prespecify noninferiority thresholds, mandate imaging surveillance, and stratify outcomes by procedural sequence, device, ablation modality, and antithrombotic regimen.
